# Comparison between the characteristics and outcomes of patients hospitalized for COVID-19 in three waves of the pandemic: a retrospective analysis

**DOI:** 10.1186/s40249-025-01389-3

**Published:** 2025-11-26

**Authors:** Bárbara Mares Porto, Daniella Nunes Pereira, Luciane Kopittke, Alisson Alves Asevedo, Angélica Gomides dos Reis Gomes, Angelinda Rezende Bhering, Beatriz Figueiredo Lima, Carla Thaís Cândida Alves da Silva, Cláudia Silva Marinho, Elayne Crestani Pereira, Evelin Paola de Almeida Cenci, Flavia Maria Borges Vigil, Gabriela Petry Crestani, Laís Mainardi dos Santos, Leila Beltrami Moreira, Marco Aurélio Reis, Maria Aparecida Camargos Bicalho, Vanessa Muller, Unaí Tupinambás, Milena Soriano Marcolino

**Affiliations:** 1 Medical School and University Hospital, Universidade Federal de Minas Gerais, Avenida Professor Alfredo Balena, 110, Santa Efigênia, Belo Horizonte, Minas Gerais CEP 30130-100 Brazil; 2 Hospital Nossa Senhora da Conceição, Av. Francisco Trein, 596, Cristo Redentor, Porto Alegre, Rio Grande Do Sul CEP 91350-200 Brazil; 3 Hospital Santa Rosália, R. Dr. Onofre, 575, Centro, Teófilo Otoni, Minas Gerais CEP 39800-022 Brazil; 4 Hospitais da Rede Mater Dei, Av. Do Contorno, 9000, Barro Preto, Belo Horizonte, Minas Gerias CEP 30110-062 Brazil; 5 Hospitais da Rede UNIMED-BH, Av. Do Contorno, 3097, Santa Efigênia. , Belo Horizonte, Minas Gerais CEP 30110-017 Brazil; 6 Orizonti - Instituto de Saúde E Longevidade, Av. José Do Patrocínio Pontes, 1355, Mangabeiras, Belo Horizonte, Minas Gerias CEP 30210-080 Brazil; 7 Hospital Santo Antônio, Praça Dr. Márcio Carvalho Lopes Filho, 501, Centro, Curvelo, Minas Gerais CEP 35790-000 Brazil; 8 Hospital Universitário Professor Edgard Santos, Universidade Federal da Bahia, R. Dr. Augusto Viana, S/N, Canela, Salvador, Bahia CEP 40110-060 Brazil; 9 Hospital SOS Cárdio, Rodovia, SC-401, 121, Itacorubi, Florianópolis, Santa Catarina CEP 88030-000 Brazil; 10 Hospital Universitário de Canoas, Universidade Federal Do Rio Grande Do Sul, Av. Farroupilha, 8001, São José, Canoas, Rio Grande Do Sul CEP 92425-900 Brazil; 11 Hospital Metropolitano Doutor Célio de Castro, Rua Dona Luiza, 311, Milionários, Belo Horizonte, Minas Gerais CEP 30620-090 Brazil; 12 Hospital Mãe de Deus, R. José de Alencar, 286, Menino Deus, Porto Alegre, Rio Grande Do Sul CEP 90880-481 Brazil; 13 Hospital Santa Cruz, Rua Fernando Abott, 174, Centro, Santa Cruz Do Sul, Rio Grande Do Sul CEP 96810-072 Brazil; 14 Hospital de Clínicas de Porto Alegre, Rua Ramiro Barcelos, 2350 Bloco A; Av. Protásio Alves, 211 - Bloco B E C, Santa Cecília, Porto Alegre, Rio Grande Do Sul CEP 90035-903 Brazil; 15 Hospital Risoleta Tolentino Neves, , Rua das Gabirobas, 01, Vila Clóris, Belo Horizonte, Minas Gerais CEP 31744-012 Brazil; 16 Fundação Hospitalar Do Estado de Minas Gerais (Rede FHEMIG), Alameda Vereador Álvaro Celso, 100, Santa Efigênia, Belo Horizonte, Minas Gerais CEP 30150-260 Brazil; 17 Hospital São Lucas da PUCRS, Av. Ipiranga, 6690, Partenon, Porto Alegre, Rio Grande Do Sul CEP 90610-001 Brazil; 18 Institute for Health Technology Assessment (IATS/ CNPq), Porto Alegre, Brazil; 19 Centro de Pesquisa Clínica, Hospital de Clínicas de Porto Alegre, Rua Ramiro Barcelos, 2350, Prédio 21 - Sala 507, Santa Cecília, Porto Alegre, Rio Grande Do Sul CEP 90035-903 Brazil

**Keywords:** COVID-19, SARS-CoV-2 variants, Pandemic, Mortality, Patient outcome assessment

## Abstract

**Background:**

COVID-19 occurred in successive waves driven by different SARS-CoV-2 variants and shaped by vaccine availability and public health measures. This study analyzes differences in clinical characteristics and outcomes of hospitalized patients across three waves in Brazil.

**Methods:**

This retrospective cohort study included adult COVID-19 patients admitted to 41 hospitals across six Brazilian states from March 2020 to August 2022. Data on demographics, clinical characteristics, and outcomes were collected from medical records and compared across three pandemic waves. Categorical variables were analyzed using Chi-square or Fisher’s exact tests, with post hoc Z tests and Bonferroni correction. Continuous variables were analyzed using Analysis of Variance (ANOVA) with Tukey’s test or Kruskal-Wallis with Dunn’s test and Bonferroni correction.

**Results:**

Among 18,632 patients, 37% were hospitalized during the first wave, 55% in the second, and 8% in the third. The median age decreased during the second wave but increased in the third (60 vs 58 vs 66 years; *P* < 0.001). A higher proportion of patients with three or more comorbidities were admitted during the third wave (15.9% vs 11.9% vs 20.6%; *P* < 0.001). Anosmia, ageusia, and fever were less frequently reported in the third wave (*P* < 0.001). Intensive care unit admissions (38.7% vs 37.1% vs 25.5%; *P* < 0.001) and in-hospital mortality (21.3% vs 23.7% vs 18.2%; *P* < 0.001) declined throughout the pandemic.

**Conclusion:**

Clinical manifestations and outcomes evolved across the pandemic waves. The third wave demonstrated fewer chemosensory symptoms, lower severity at admission, and reduced mortality, despite an older and more comorbid patient population.

**Graphical Abstract:**

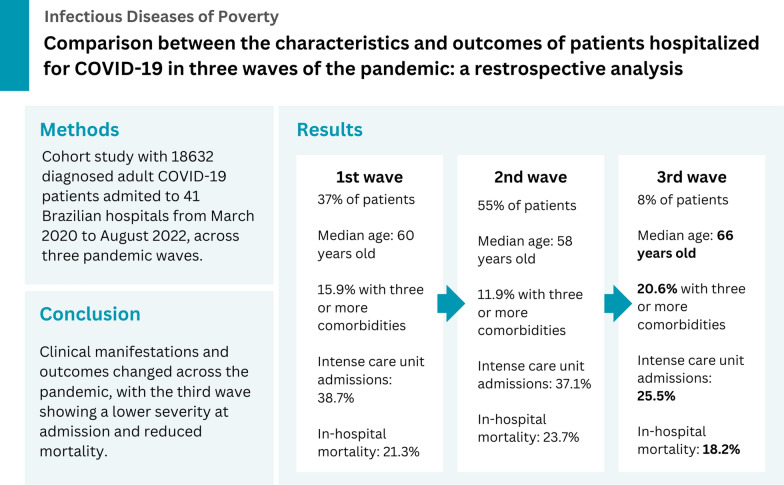

**Supplementary Information:**

The online version contains supplementary material available at 10.1186/s40249-025-01389-3.

## Background

Throughout the COVID-19 pandemic, new SARS-CoV-2 variants emerged, each prevailing during a certain time period and defining the pandemic waves that were observed worldwide [1]. Even in different countries, such waves presented many similarities, such as the period of occurrence, clinical characteristics and outcomes of those who were infected by the SARS-CoV-2, and governmental public policies to stop the spread of the disease. Their major differences between the waves are believed to be related to the SARS-CoV-2 variant circulating at the time, as well as the availability of vaccines for the population [1, 2].

For instance, the first COVID-19 wave began in Wuhan, China, after the discovery of the virus in December 2019 and ended in mid-November 2020 [1]. During the first wave, the world experienced the initial dissemination of the SARS-CoV-2, while transmission mechanisms, treatment and prevention methods were still uncertain or not well understood. By the end of 2020, the virus had undergone mutations that initiated subsequent pandemic waves [1].

The second wave was characterized by the predominance of the Delta variant of the SARS-CoV-2, beginning at a time in which vaccination was still scarce and lockdowns were still enforced in many countries [3]. The Delta variant prevailed from November 2020 until December 2021, causing a high number of cases and deaths by COVID-19 worldwide [1]. The Omicron variant, first identified in November 2021, quickly spread and became the dominant strain in many countries worldwide, characterizing the third wave [4]. This wave lasted from December 2021 until the end of 2022 [1]. Subsequently, with increased knowledge about the virus, improved symptom management, greater adherence to prevention strategies, broader access to antivirals and vaccines, and the containment of the Omicron variant, the number of cases and deaths dropped drastically, leading to the end of the COVID-19 emergency, as announced by the World Health Organization (WHO) in May 2023 [5, 6].

Despite the widespread availability of vaccines and a notable reduction in COVID-19 cases in recent years, the disease continues to pose significant challenges globally. Brazil has reported over 38 million confirmed cases, and remains one of the countries most affected by the pandemic [7]. With ongoing concerns about emerging variants and their implications for public health, this study aims to examine the differences in clinical characteristics and outcomes of patients hospitalized during three distinct waves of COVID-19 in Brazil. By analyzing these changes, we seek to provide critical insights that can inform resource allocation and enhance public health strategies to address the dynamic nature of the pandemic.

## Methods

### Study design

This study is part of a multicentric retrospective cohort, entitled the Brazilian COVID-19 Registry, described in detail previously [8]. The study protocol was approved by the National Commission for Research Ethics (CAAE: 30,350,820.5.1001.0008). The need for individual informed consent was waived and the analysis was performed using unidentified data from medical records only.

**Fig. 1:**
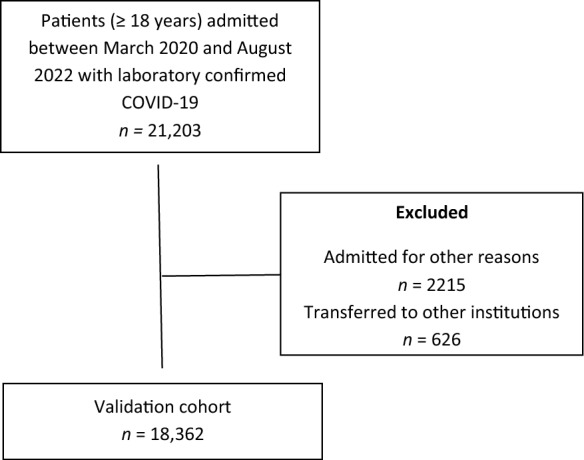
Flowchart of patient selection for the study

The present study included adult patients (≥ 18 years), with laboratory-confirmed COVID-19, admitted between March 2020 and August 2022 in 41 hospitals in six Brazilian states: Bahia, Minas Gerais, Pernambuco, Rio Grande do Sul, Santa Catarina and São Paulo (Table S5). In accordance with World Health Organization guidance, COVID-19 diagnosis was confirmed primarily by RT-PCR on nasopharyngeal or oropharyngeal swabs. Serological testing for IgM antibodies was used only in cases where RT-PCR was unavailable due to temporary reagent shortages during the early phase of the COVID-19 pandemic [8, 9]. We excluded patients who were transferred to other institutions, as well as those in whom the manifestation of COVID-19 occurred only after hospitalization (i.e., those admitted for reasons unrelated to COVID-19) (Fig. 1). Hospitals were invited to join the Brazilian COVID-19 Registry through various channels, including social media, radio and the website of the National Institute of Science and Technology for Health Technology Assessment (Instituto de Avaliação de Tecnologias em Saúde – IATS), as previously described [8].

This manuscript adheres to the Strengthening the Reporting of Observational Studies in Epidemiology (STROBE) guidelines. [10]

### Data collection

Demographic data, clinical characteristics, and outcomes were collected from the medical records by trained researchers to the Research Electronic Data Capture (REDCap®) electronic platform, hosted at the Telehealth Center of the University Hospital from Universidade Federal de Minas Gerais [11–13]. In order to identify possible inconsistencies, all acquired information underwent a periodic automatic validation using a code developed using the R program for statistical computing (version 4.0.2). All inconsistencies were sent to each center, which checked the data and made the necessary corrections, guaranteeing data quality.

### Outcomes

The primary outcome was in-hospital mortality. Secondary outcomes included: intensive care unit (ICU) admission, need for mechanical ventilation, renal replacement therapy (RRT) thromboembolic event, nosocomial infection, acute heart failure, septic shock and need for vasopressor.

### Statistical analysis

In the descriptive analysis, absolute and relative frequencies were used to represent demographic and clinical data. Continuous variables were represented by median and interquartile range (IQR) or mean and standard deviation, according to the Kolmogorov Smirnov test to verify data normality. As for counts, numbers and percentages were used.

For the purpose of this analysis, the COVID-19 pandemic was divided in three waves, based on three peaks of death extracted from the public panel of the Brazilian Ministry of Health: (i) the first from 10 March 2020 to 14 November 2020, (ii) the second from 15 November 2020 to 25 December 2021, (iii) and, the third from 26 December 2021 to 03 August 2022 [1]. Comparisons among the three pandemic waves were conducted using the Chi-Square test or Fisher’s exact test for categorical variables, with post hoc comparisons performed using the Z test with Bonferroni correction. For continuous variables, normally distributed data were analyzed using Analysis of Variance (ANOVA) followed by Tukey’s test, while non-normally distributed data were analyzed using the Kruskal-Wallis test followed by Dunn’s test with Bonferroni correction. No regression analysis was performed.

Results with a significance level of 5% were considered statistically significant. Statistical analysis was performed with the R program for statistical computing version 4.0.2 (R Foundation for Statistical Computing, Vienna, Austria) and IBM SPSS Statistics for Windows and Macintosh version 25 (IBM Corp., Armonk, NY, USA).

## Results

Among 18,362 patients with COVID-19 admitted to participating hospitals, 6799 (37%) were diagnosed in the first wave, 10,089 (55%) in the second and 1474 (8%) in the third. All comparisons presented below are based on univariate statistical analyses.

### Demographic and baseline characteristics

The median age decreased in the second wave compared to the first (60 vs 58 years; *P* < 0.001) and increased again in the third wave compared to both the first (66 vs 60 years; *P* < 0.001) and second waves (66 vs 58 years; *P* < 0.001). Throughout the pandemic, the number of hospitalized women increased, with a higher proportion in the third wave compared to both the first and second waves (*P* < 0.001).

There was a lower frequency of patients admitted with three or more comorbidities in the second wave compared to the first (11.9% vs 15.4%; *P* < 0.001), and a higher frequency in the third wave compared to both the first (20.6% vs 15.4%; *P* < 0.001) and second waves (20.6% vs 11.9%; *P* < 0.001). Lifestyle risk factors such as alcohol use disorder, current smoking, and illicit drug use were more frequent in the third wave compared to both the first and second waves (*P* < 0.001 for all). Regarding functionality, there was no data collected in the first wave. However, there was an increase in frail patients admitted in the third wave compared to the second, with a reduction of robust patients (86.9% vs 51.0%, *P* < 0.001). Also in the third wave, there was a greater number of patients on prior use of immunosuppressants (1.3% vs 1.6% vs 5.3%, *P* < 0.001).

With regards to the clinical characteristics at hospital presentation, tachycardia was less frequent in the second and third waves compared to the first (16.9% and 16.0% vs 22.6%; *P* < 0.001), with no difference between the second and third waves. Tachypnea was more frequent in the second wave compared to the first (25.7% vs 21.1%; *P* < 0.001) and markedly less frequent in the third compared to both previous waves (11.8% vs 21.1% and 25.7%; *P* < 0.001). Regarding oxygenation status, patients in the second wave presented with more severe impairment compared to both the first and third waves, with a higher frequency of SpO₂/FiO₂ ≤ 150 and a lower frequency of SpO₂/FiO₂ > 315 (*P* < 0.001), while the first and third waves did not differ significantly from each other.

Table 1 shows demographic data, clinical characteristics, and lifestyle habits of COVID-19 patients according to the period of infection.

**Table 1:** Clinical characteristics of COVID-19 patients, 41 Brazilian hospitals, March 2020–August 2022

Characteristics	1st wave *n (%)*	2nd wave *n (%)*	3rd wave *n (%)*	*P-value*
*n*	6799	10,089	1474	
Mean age (years)*	60^a^ (16.7)	58^b^ (15.9)	66^c^ (18.3)	< 0.001
Women	3095^a^ (45.5%)	4699^a^ (46.6%)	744^b^ (50.5%)	< 0.001
*Cardiovascular diseases*				
Hypertension	3710^a^ (54.6%)	5236^b^ (51.9%)	822^a^ (55.8%)	< 0.001
Heart failure	447^a^ (6.6%)	412^b^ (4.1%)	170^c^ (11.5%)	< 0.001
Coronary artery disease	375^a^ (5.5%)	391^b^ (3.9%)	115^c^ (7.8%)	< 0.001
Ischemic stroke	257^a^ (3.8%)	249^b^ (2.5%)	119^c^ (8.1%)	< 0.001
Atrial fibrillation or flutter	228^a^ (3.4%)	192^b^ (1.9%)	79^c^ (5.4%)	< 0.001
Chagas disease	23^a^ (0.3%)	30^a^ (0.3%)	8^a^ (0.5%)	0.309
Respiratory diseases				
Asthma	426^a^ (6.3%)	548^a^ (5.4%)	93^a^ (6.3%)	0.053
COPD	446^a^ (6.6%)	419^b^ (4.2%)	196^c^ (13.3%)	< 0.001
Pulmonary fibrosis	38^a^ (0.6%)	24^b^ (0.2%)	16^a^ (1.1%)	< 0.001
Metabolic diseases				
Diabetes mellitus	1985^a^ (29.2%)	2544^b^ (25.2%)	453^a^ (30.7%)	< 0.001
Obesity	1222^a^ (18.0%)	1958^a^ (19.4%)	148^b^ (10.0%)	< 0.001
Other conditions				
Active cancer	367^a^ (5.4%)	284^b^ (2.8%)	143^c^ (9.7%)	< 0.001
Chronic kidney disease	377^a^ (5.5%)	364^b^ (3.6%)	128^c^ (8.7%)	< 0.001
Rheumatological disease	123^a^ (1.8%)	192^a^ (2.0%)	56^b^ (3.0%)	< 0.001
HIV infection	67^a^ (1.0%)	59^b^ (0.6%)	21^a^ (1.9%)	< 0.001
Previous transplant	53^a^ (0.8%)	69^a^ (0.7%)	50^b^ (3.4%)	< 0.001
Cirrhosis	40^a^ (0.6%)	27^b^ (0.3%)	14^a^ (0.9%)	< 0.001
Number of comorbidities				< 0.001
0	1973^a^ (29.0%)	3401^b^ (33.7%)	349^c^ (23.7%)	
1	2012^a^ (29.6%)	3160^a^ (31.3%)	426^a^ (28.9%)	
2	1765^a^ (26.0%)	2323^b^ (23.0%)	395^a^ (26.8%)	
3 or more	1049^a^ (15.4%)	1205^b^ (11.9%)	304^c^ (20.6%)	
Functionality		Non-missing cases (*n* = *6293*)	Non-missing cases (*n* = *679*)	< 0.001
Robust	NA	5468^b^ (86.9%)	346^a^ (51.0%)	
Vulnerable or mildly frail	NA	486^b^ (7.7%)	152^a^ (22.4%)	
Moderately frail	NA	140^b^ (2.2%)	68^a^ (10%)	
Severely or very severely frail	NA	188^b^ (3.0%)	113^a^ (16.6%)	
Terminally ill	NA	11^a^ (0.2%)	0^a^ (0.0%)	
Lifestyle habits				
Alcohol use disorder	272^a^ (4.0%)	547^b^ (5.4%)	101^b^ (6.9%)	< 0.001
Current smoking	250^a^ (3.7%)	316^a^ (3.1%)	136^b^ (9.2%)	< 0.001
Illicit drug use	44^a^ (0.6%)	48^a^ (0.5%)	19^b^ (1.3%)	< 0.001
Previous smoking	1086^a^ (16.0%)	1419^b^ (14.1%)	277^c^ (18.8%)	< 0.001
Clinical characteristics at presentation				
GCS < 15	935^a^ (13.8%)	478^b^ (4.7%)	178^a^ (12.1%)	< 0.001
Invasive mechanical ventilation	484^a^ (7.1%)	93^b^ (1.1%)	2^c^ (0.2%)	< 0.001
Tachycardia	1472^a^ (22.6%)	1657^b^ (16.9%)	231^b^ (16.0%)	< 0.001
Tachypnea	1178^a^ (21.1%)	2242^b^ (25.7%)	148^c^ (11.8%)	< 0.001
SpO_2_/FiO_2_	Non-missing cases (*n* = *3189*)	Non-missing cases (*n* = *7145*)	Non-missing cases (*n* = *758*)	< 0.001
≤ 150	787^a^ (24.7%)	2030^b^ (28.4%)	129^c^ (17%)	
150–235	232^a^ (7.3%)	625^b^ (8.7%)	56^a,b^ (7.4%)	
235–315	344^a^ (20.2%)	1905^b^ (26.7%)	181^a,b^ (23.9%)	
> 315	1526^a^ (47.9%)	2585^b^ (36.2%)	392^a^ (51.7%)	

This table summarizes the demographic data, clinical characteristics, and lifestyle habits of patients hospitalized with COVID-19 in 41 Brazilian hospitals between March 2020 and August 2022, according to the period of infection. Numbers are presented as n (%), compared by Pearson's Chi-squared test or Fisher's exact test, if necessary, followed by Z test of proportion comparison with Bonferroni’s correction. ^a,b,c^ If the *P*-value is significant, the superscript letters “a”, “b” and “c” inform in which comparison there is difference. If both groups have the same superscript letter, there is not statistically significance in that comparison. When each group has a different letter, there is a significant difference

^*^Mean and standard deviation (SD), Analysis of Variance (ANOVA) followed by Tukey’s test for multiple comparison

*COPD* chronic obstructive pulmonary disease, *GCS* Glasgow Coma Scale, *FiO*_*2*_ fraction of inspired oxygen, *SpO*_*2*_ peripheral oxygen saturation; *NA* not applicable; *HIV* Human Immunodeficiency Virus; tachycardia > 100; tachypnea > 20. 1st wave:10 March 2020 to 14 November 2020; 2nd wave: 15 November 2020 to 25 December 2021; 3rd wave: 26 December 2021 to 03 August 2022

### In-hospital outcomes

There were no significant differences in septic shock, dialysis and mechanical ventilation between the first and second wave, but there was a notable reduction in the third one: for septic shock 13.4% vs 14.1% vs 10.1%, *P* < 0.001; for dialysis 11.3% vs 11.2% vs 6.4%, *P* < 0.001; for mechanical ventilation 29.3% vs 28.7% vs 14.8%, *P* < 0.001. Thromboembolic events were more frequent in the second wave compared to the first (6.3% vs 5.2%; *P* = 0.02) and then decreased markedly in the third (1.8%; *P* < 0.001 vs both previous waves). In-hospital mortality followed a similar pattern, being higher in the second compared to the first wave (23.7% vs 21.3%; *P* = 0.001) and subsequently lower in the third compared to both (18.2%; *P* < 0.001). The frequency of admission to the intensive care unit (38.7% vs 37.1% vs 25.5%, *P* < 0.001) and need for vasopressors (5.5% vs 3.9% vs 3.1%, *P* < 0.001) reduced throughout the pandemic, with the lowest value found in the third wave.

These results are summarized in Table 2.

**Table 2:** Clinical outcomes of COVID-19 patients, 41 Brazilian hospitals, March 2020–August 2022, by period of infection

Outcomes	1st wave *n (%)*	2nd wave *n (%)*	3rd wave *n %*	*P-value*
*n*	6799	10,089	1474	
In-hospital mortality	1443^a^ (21.3%)	2379^b^ (23.7%)	268^c^ (18.2%)	< 0.001
ICU admission	2628^a^ (38.7%)	3735^b^ (37.1%)	375^c^ (25.5%)	< 0.001
Invasive mechanical ventilation	2628^a^ (29.3%)	3735^a^ (28.7%)	218^b^ (14.8%)	< 0.001
Renal Replacement Therapy	765^a^ (11.3%)	1124^a^ (11.2%)	94^b^ (6.4%)	< 0.001
Thromboembolic events	351^a^ (5.2%)	631^b^ (6.3%)	26^c^ (1.8%)	< 0.001
Nosocomial infection	697^a^ (10.3%)	1547^b^ (15.3%)	153^a^ (10.4%)	< 0.001
Acute heart failure	179^a^ (2.6%)	193^b^ (1.9%)	56^c^ (3.8%)	< 0.001
Septic shock	910^a^ (13.4%)	1419^a^ (14.1%)	148^b^ (10.1%)	< 0.001
Vasopressors	371^a^ (5.5%)	391^b^ (3.9%)	45^b^ (3.1%)	< 0.001

Numbers are presented as n (%), compared by Pearson's Chi-squared test or Fisher's exact test, if necessary, followed by Z test of proportion comparison with Bonferroni’s correction. ^a,b,c^ If the *P*-value is significant, the superscript letters “a”, “b” and “c” inform in which comparison there is difference. If both groups have the same superscript letter, there is not statistically significance in that comparison. When each group has a different letter, there is a significant difference

1st wave:10 March 2020 to 14 November 2020; 2nd wave: 15 November 2020 to 25 December 2021; 3rd wave: 26 December 2021 to 03 August 2022

### COVID-19 associated symptoms

In the third wave, anosmia was significantly less common compared to both the first and second waves (2.1% vs 10.9% and 10.0%; *P* < 0.001), as was ageusia (2.2% vs 7.5% and 8.9%; *P* < 0.001). Fever was also less frequent in the third wave compared to both the first (41.7% vs 56.8%; *P* < 0.001) and second waves (41.7% vs 50.4%; *P* < 0.001). Conversely, neurological manifestations were more frequent in the third wave compared to both previous waves (4.1% vs 2.4% and 1.8%; *P* < 0.001).

Detailed data on symptoms are presented in Table S1.

### Therapies

The use of systemic corticosteroids increased from 70.4% in the first wave to 92.5% in the second, and then decreased to 75.6% in the third (*P* < 0.001). Specific therapies against were overall infrequently used, but tocilizumab followed the same pattern, with use rising from 0.1% in the first wave to 1.5% in the second and then decreasing to 0.7% in the third (*P* < 0.001). Anticoagulant use was high in the first two waves (89.5% and 92.1%) but significantly reduced in the third (82.6%; *P* < 0.001).

Therapeutic data are summarized in Table S2.

### Vaccination status

There were no vaccinated patients in the first wave, as vaccines only became available in Brazil from January 2021. A substantial amount of missing data was noted, particularly in the second wave (72.0%) compared to the third (45.9%). Among those with available information, vaccination coverage increased significantly from the second to the third wave (9.9% vs 46.0%; *P* < 0.001). In the second wave, most vaccinated patients had received one (42.5%) or two (49.4%) doses, whereas in the third wave a much higher proportion had received three doses (37.6% vs 2.2%; *P* < 0.001). Regarding vaccine type, Coronavac predominated in the second wave, while in the third wave the distribution was more balanced among Pfizer, Coronavac, and AstraZeneca.

Additional details on vaccination are shown in Tables S3 and S4.

## Discussion

This study reveals notable differences in clinical characteristics and outcomes among patients across the three COVID-19 waves in a large Brazilian cohort. Consistent with previous studies that have comparatively analyzed pandemic waves, there was a decrease in severity throughout the pandemic [14–17]. In our analysis, admission to intensive care units and in-hospital mortality reduced throughout the pandemic, reaching the lowest levels in the third wave. Despite the older age and the higher frequency of comorbidities during the third wave, patients were admitted in a less severe condition when compared to the other waves. They exhibited a lower incidence of tachycardia, tachypnea, and need for vasopressors, and had a lower frequency of severe outcomes, like renal replacement therapy, thromboembolic events, septic shock and mechanical ventilation. Notably, the prevalence of smell and taste disorders (e.g., anosmia and ageusia) was also lower in the third wave, consistent with the predominance of the Omicron variant. This reduction in chemosensory changes with newer variants has been observed in other studies [15, 18]. These trends can be attributed to multiple factors, including viral mutations, increasing knowledge about the disease, and the gradual build-up of immunity through infection and vaccination. These factors are believed to have collectively mitigated the severity and incidence of worse clinical outcomes.

Previous research has highlighted differences in severity between the SARS-CoV-2 variants [14–17]. Delta variant was first identified at the end of 2020 and had since been the most prevalent variant worldwide until the emergence of the Omicron [14, 15]. In Brazil, the Gamma variation was predominant in the first stage of the second wave, with the subsequent appearance of the Delta variant [1]. Delta was more transmissible and was associated with a higher risk of severe disease and hospitalization, demonstrated in studies of other countries, like India, United Kingdom and South Africa [14–17]. The Omicron variant rapidly spread around the globe, replacing all previously dominant circulating SARS-CoV-2 viruses, being dominant in the third wave. Observational data from multiple studies suggest that this variant has higher transmissibility, probably due intrinsic characteristics and immune evasion, but has lower risk of severe infection. The reduced risk for severe disease may reflect partial protection conferred by prior infection or vaccination [4, 14, 15, 17].

Brazil's COVID-19 vaccination campaign began in January 2021, covering more vulnerable groups, such as the elderly, those with comorbidities, vulnerable population and healthcare workers. A significant increase in vaccination coverage occurred only in the second half of 2021, reaching 50% coverage around September 2021—near the end of the second wave, which in our study extended until December 2021 [1]. Although vaccination had already started during the second wave, coverage was still limited, and most individuals had received only one or two doses, with the general population largely unprotected. This period also coincided with the predominance of the Gamma and later Delta variants, which have been associated with higher transmissibility, increased disease severity, and higher risk of thromboembolic complications [14–17]. In addition, the second wave placed extreme pressure on the healthcare system in Brazil, potentially leading to delays in diagnosis and treatment, and contributing to the higher in-hospital mortality and thromboembolic events observed in our cohort compared to the first wave.

The initial vaccine rollout primarily included CoronaVac (Sinovac Biotech) and ChAdOx1 nCov-19 (AstraZeneca/Oxford University) vaccines. The BNT162b2 (Pfizer-BioNTech) and Ad26.Cov2.S (Johnson & Johnson-Janssen) vaccines were incorporated later in the campaign (May and June 2021, respectively). In the third wave, around 85% of the patients who answered about vaccination had received at least one dose of vaccine, with the majority receiving 2 or more doses. These vaccines have significantly reduced the risk of severe or critical COVID-19 and have been associated with substantial reductions in COVID-19-related hospitalizations and deaths [19, 20]. A study using data from the same Brazilian COVID-19 Registry performed multivariate analyses specifically focused on the effectiveness of COVID-19 vaccines and confirmed an independent association between vaccination and reduced mortality and ICU admission, even after adjusting for potential confounders [21]. These findings support the hypothesis that increased vaccination coverage contributed to the milder clinical profiles and improved outcomes observed in the third wave.

With regards to therapies used against COVID-19, the use of systemic corticosteroids was greater in the second wave, likely due to increasing knowledge, experience and a greater case severity. This explanation can also be applied to the increase in the use of non-invasive ventilation in the second wave. These findings were seen in a retrospective study conducted at the University Hospital of the *Universidade Federal de São Paulo*, Brazil [22]. Specific therapies, like immunomodulators and Remdesvir were little used overall, as they were absent or scarcely available in both public and private hospitals at the time.

This study has some limitations. First, it is a retrospective analysis and some variables were not consistently collected across all waves. Second, no regression analysis was performed; therefore, the study design does not allow us to infer causality from the associations observed. Third, despite being a large cohort, including five Brazilian states, may not represent the entire population. Fourth, infection with the variants described were assigned based on their prevalence in the Brazilian population at the time and not on virus genotyping. Fifth, about vaccination, there was a considerable amount of missing values. Additionally, antiviral therapies were scarcely available in both public and private hospitals during the study period, which limited their relevance in outcome prediction. Given the large sample size across the three pandemic waves, most comparisons had adequate statistical power to detect even modest differences. However, for some variables with very low frequency, such as pulmonary fibrosis, the ability to detect statistically significant differences may have been limited. In such cases, the absence of statistical significance does not necessarily rule out the possibility of clinically relevant differences and should be interpreted with appropriate caution.

## Conclusion

The clinical manifestations and outcomes changed between the three COVID-19 waves. There was a decrease of incidence of chemosensory changes in the third wave. Notably, severe outcomes and mortality decreased throughout the pandemic, with a marked reduction in the third wave, even though patients in this wave were older and had more comorbidities. These results suggest that changes in the epidemiological context — including higher vaccination coverage, improved clinical management, and the circulation of different variants — may have contributed to the observed patterns. While causality cannot be inferred due to the observational study design, the findings provide relevant evidence from a large multicenter cohort in a middle-income country, helping to better understand the evolving burden of COVID-19.

## Supplementary Information

Supplementary file 1.

## Data Availability

Data is available upon reasonable request.

## References

[1] Moura EC, Cortez-Escalante J, Cavalcante FV, Barreto ICHC, Sanchez MN, Santos LMP. COVID-19: temporal evolution and immunization in the three epidemiological waves, Brazil, 2020–2022. Rev Saude Publica. 2022;56:105. 10.11606/s1518-8787.2022056004907. 36515307 10.11606/s1518-8787.2022056004907 PMC9749655

[2] Chi WY, Li YD, Huang HC, Chan TEH, Chow SY, Su JH, et al. COVID-19 vaccine update: vaccine effectiveness, SARS-CoV-2 variants, boosters, adverse effects, and immune correlates of protection. J Biomed Sci. 2022;29(1):82. 10.1186/s12929-022-00853-8. 36243868 10.1186/s12929-022-00853-8 PMC9569411

[3] Miyashita K, Hozumi H, Furuhashi K, Nakatani E, Inoue Y, Yasui H, et al. Changes in the characteristics and outcomes of COVID-19 patients from the early pandemic to the delta variant epidemic: a nationwide population-based study. Emerg Microbes Infect. 2023;12(1):2155250. 10.1080/22221751.2022.2155250. 36469641 10.1080/22221751.2022.2155250 PMC9788709

[4] Tian D, Sun Y, Xu H, Ye Q. The emergence and epidemic characteristics of the highly mutated SARS-CoV-2 Omicron variant. J Med Virol. 2022;94(6):2376–83. 10.1002/jmv.27643. 35118687 10.1002/jmv.27643 PMC9015498

[5] Araf Y, Akter F, Tang YD, Fatemi R, Parvez MSA, Zheng C, et al. Omicron variant of SARS-CoV-2: genomics, transmissibility, and responses to current COVID-19 vaccines. J Med Virol. 2022;94(5):1825–32. 10.1002/jmv.27588. 35023191 10.1002/jmv.27588 PMC9015557

[6] United Nations. WHO chief declares end to COVID-19 as a global health emergency. United Nations News. 2023 May 5. https://news.un.org/en/story/2023/05/1136367. Accessed 1 Apr 2024.

[7] Ministério da Saúde, Governo do Brasil. Coronavírus Brasil. https://covid.saude.gov.br/. Accessed 1 Apr 2024.

[8] Marcolino MS, Ziegelmann PK, Souza-Silva MVR, Nascimento IJB, Oliveira LM, Monteiro LS, et al. Clinical characteristics and outcomes of patients hospitalized with COVID-19 in Brazil: results from the Brazilian COVID-19 registry. Int J Infect Dis. 2021;107:300–10. 10.1016/j.ijid.2021.01.019. 33444752 10.1016/j.ijid.2021.01.019 PMC7801187

[9] World Health Organization. Diagnostic testing for SARS-CoV-2: interim guidance. 2020. https://www.who.int/publications/i/item/diagnostic-testing-for-sars-cov-2. Accessed 1 Apr 2024.

[10] von Elm E, Altman DG, Egger M, Pocock SJ, Gøtzsche PC, Vandenbroucke JP, et al. The strengthening the reporting of observational studies in epidemiology (STROBE) statement: guidelines for reporting observational studies. J Clin Epidemiol. 2008;61(4):344–9. 10.1016/j.jclinepi.2007.11.008. 18313558 10.1016/j.jclinepi.2007.11.008

[11] Harris PA, Taylor R, Thielke R, Payne J, Gonzalez N, Conde JG. Research electronic data capture (REDCap): a metadata-driven methodology and workflow process for providing translational research informatics support. J Biomed Inform. 2009;42(2):377–81. 10.1016/j.jbi.2008.08.010. 18929686 10.1016/j.jbi.2008.08.010 PMC2700030

[12] Harris PA, Taylor R, Minor BL, Elliott V, Fernandez M, O’Neal L, et al. REDCap Consortium The REDCap consortium: building an international community of software platform partners. J Biomed Inform. 2019;95:103208. 10.1016/j.jbi.2019.103208. 31078660 10.1016/j.jbi.2019.103208 PMC7254481

[13] Marcolino MS, Figueira RM, Dos Santos JPA, Cardoso CS, Ribeiro AL, Alkmim MB. The experience of a sustainable large-scale Brazilian telehealth network. Telemed J E-Health. 2016;22(11):899–908. 10.1089/tmj.2015.0234. 27167901 10.1089/tmj.2015.0234

[14] Abdullah F, Myers J, Basu D, Tintinger G, Ueckermann V, Mathebula M, et al. Decreased severity of disease during the first global Omicron variant COVID-19 outbreak in a large hospital in Tshwane, South Africa. Int J Infect Dis. 2022;116:38–42. 10.1016/j.ijid.2021.12.357. 34971823 10.1016/j.ijid.2021.12.357 PMC8713416

[15] Menni C, Valdes AM, Polidori L, Antonelli M, Penamakuri S, Nogal A, et al. Symptom prevalence, duration, and risk of hospital admission in individuals infected with SARS-CoV-2 during periods of Omicron and Delta variant dominance: a prospective observational study from the ZOE COVID study. Lancet. 2022;399(10335):1618–24. 10.1016/S0140-6736(22)00327-0. 35397851 10.1016/S0140-6736(22)00327-0 PMC8989396

[16] Singh S, Sharma A, Gupta A, Joshi M, Aggarwal A, Soni N, et al. Demographic comparison of the first, second and third waves of COVID-19 in a tertiary care hospital at Jaipur. India Lung India. 2022;39(6):525–31. 10.4103/lungindia.lungindia_265_22. 36629231 10.4103/lungindia.lungindia_265_22 PMC9746281

[17] Nyberg T, Ferguson NM, Nash SG, Webster HH, Flaxman S, Andrews N, et al. Comparative analysis of the risks of hospitalization and death associated with SARS-CoV-2 Omicron (B.1.1.529) and Delta (B.1.617.2) variants in England: a cohort study. Lancet. 2022;399(10332):1303–12. 10.1016/S0140-6736(22)00462-7. 35305296 10.1016/S0140-6736(22)00462-7 PMC8926413

[18] Coelho DH, Reiter ER, French E, Costanzo RM. Decreasing incidence of chemosensory changes by COVID-19 variant. Otolaryngol Head Neck Surg. 2023;168(4):704–6. 10.1177/01945998221097656. 35503739 10.1177/01945998221097656 PMC9630171

[19] Thompson MG, Stenehjem E, Grannis S, Ball SW, Naleway AL, Ong TC, et al. Effectiveness of COVID-19 vaccines in ambulatory and inpatient care settings. N Engl J Med. 2021;385(15):1355–71. 10.1056/NEJMoa2110362. 34496194 10.1056/NEJMoa2110362 PMC8451184

[20] Zheng C, Shao W, Chen X, Zhang B, Wang G, Zhang W. Real-world effectiveness of COVID-19 vaccines: a literature review and meta-analysis. Int J Infect Dis. 2022;114:252–60. 10.1016/j.ijid.2021.11.009. 34800687 10.1016/j.ijid.2021.11.009 PMC8595975

[21] de Moraes EV, Pires MC, Costa AAA, et al. Comprehensive statistical analysis reveals significant benefits of COVID-19 vaccination in hospitalized patients: propensity score, covariate adjustment, and feature importance by permutation. BMC Infect Dis. 2024;24:1052. 10.1186/s12879-024-09865-1. 39333931 10.1186/s12879-024-09865-1 PMC11428431

[22] Freitas DHM, Costa ELV, Zimmermann NA, Gois LSO, Anjos MVA, Lima FG, et al. Temporal trends of severity and outcomes of critically ill patients with COVID-19 after the emergence of variants of concern: a comparison of two waves. PLoS ONE. 2024;19(3):e0299607. 10.1371/journal.pone.0299607. 38452031 10.1371/journal.pone.0299607 PMC10919739

