# Supplementary material for: Comparison between the characteristics and outcomes of patients hospitalized for COVID-19 in three waves of the pandemic: a retrospective analysis
Source: Infect Dis Poverty. 2025 Nov 26;14:119. doi: 10.1186/s40249-025-01389-3 (PMC12648880; doi:10.1186/s40249-025-01389-3)
Supplement: Supplementary file 1 [file 40249_2025_1389_MOESM1_ESM.docx]

**Supplementary Files**

**Table S1.** Admission symptoms, laboratory findings, and medications in use of COVID-19 patients, 41 Brazilian hospitals, March 2020–August 2022

| **Characteristics** | **1st wave**  ***n(%)*** | **2nd wave**  ***n(%)*** | **3rd wave**  ***n(%)*** | ***P-value*** |
| --- | --- | --- | --- | --- |
| ***n*** | **6799** | **10,089** | **1474** |  |
| *Symptoms* |  |  |  |  |
| Adynamia | 1678^a^ (24.7%) | 2197^b^ (21.8%) | 263^c^ (17.8%) | <0.001 |
| Ageusia | 512^a^ (7.5%) | 893^b^ (8.9%) | 33^c^ (2.2%) | <0.001 |
| Anosmia | 741^a^ (10.9%) | 1012^a^ (10.0%) | 31^b^ (2.1%) | <0.001 |
| Cough | 857^a^ (12.6%) | 6645^b^ (65.9%) | 952^b^ (64.6%) | <0.001 |
| Diarrhea | 976^a^ (14.4%) | 1611^b^ (16%) | 10.5^c^ (8.3%) | <0.001 |
| Dyspnea | 4083^a^ (60.1%) | 6781^b^ (67.2%) | 834^c^ (56.6%) | <0.001 |
| Fever | 3864^a^ (56.8%) | 5086^b^ (50.4%) | 615^c^  (41.7%) | <0.001 |
| Hyporexia | 745^a^ (11.0%) | 1459^b^ (14.5%) | 241^b^ (16.4%) | <0.001 |
| Myalgia | 2128^a^ (27.7%) | 3458^b^ (30.8%) | 319^c^ (13.9%) | <0.001 |
| Nausea/vomiting | 805^a^ (11.8%) | 1321^b^ (13.1%) | 156^a^ (10.6%) | <0.001 |
| Neurological manifestations | 161^a^ (2.4%) | 183^b^ (1.8%) | 61^c^ (4.1%) | <0.001 |
| Rhinorrhea | 870^a^ (12.8%) | 1203^a^ (11.9%) | 228^b^ (15.5%) | <0.001 |
| *Laboratory findings* |  |  |  |  |
| Hemoglobin (g/dL)* | 12.9^a^ ±2.2 | 13.0^b^ ±2.0) | 11.8^c^ ±2.4 | <0.001 |
| Leucocytes (cels/mm3)** | 7100^a^ (5200-9900) | 7910^b^ (5830-10760) | 8030^b^ (5800-11242) | <0.001 |
| Neutrophils (cels/mm^3^)** | 5183^a^ (3479-7744) | 6120^b^ (4272-8760) | 5833^c^  (3762-8510) | <0.001 |
| Lymphocytes (cels/mm^3^)** | 1041^a^ (713-1480) | 960^b^ (668-1352) | 1120^c^ (699-1674) | <0.001 |
| Platelets (cels/mm^3^)* | 215872^a^ (93367) | 224779^b^ (94839) | 220806^a,b^ (106423) | <0.001 |
| Bilirubin (mg/dL)** | 0,45^a^ (0.30-0.67) | 0,41^a^ (0.30-0.60) | 0,5^b^ (0.32-0.80) | <0.001 |
| *Medications in use* |  |  |  |  |
| Oral anticoagulant | 461^a^ (6.8%) | 378^b^ (3.7%) | 89^a^ (6.0%) | <0.001 |
| Inhalatory corticoids | 212^a^ (3.1%) | 153^b^ (1.5%) | 40^a^ (2.7%) | <0.001 |
| Oral corticoid | 176^a^ (2.6%) | 182^b^ (1.8%) | 93^c^ (6.3%) | <0.001 |
| Immunosuppressants | 88^a^ (1.3%) | 164^a^ (1.6%) | 78^b^ (5.3%) | <0.001 |
| This table presents the distribution of symptoms, laboratory findings, and medications in use at hospital admission of COVID-19 patients from 41 Brazilian hospitals, stratified by period of infection. Numbers are presented as n (%), compared by Pearson's Chi-squared test or Fisher's exact test, if necessary, followed by Z test of proportion comparison with Bonferroni’s correction. ^a,b,c^ If the *P*-value is significant, the superscript letters “a”, “b” and “c” inform in which comparison there is difference. If both groups have the same superscript letter, there is not statistically significance in that comparison. When each group has a different letter, there is a significant difference.  *Mean and standard deviation (SD), Analysis of Variance (ANOVA) followed by Tukey’s test for multiple comparison.  ** Median and interquartile interval (25^th^ and 75^th^ percentile) , Kruskal-Wallis followed by Dunn’s test with Bonferroni’s correction for multiple comparison.  1st wave:10 March 2020 to 14 November 2020; 2nd wave: 15 November 2020 to 25 December 2021; 3rd wave: 26 December 2021 to 03 August 2022. | | | | |

| **Table S2.** Therapies administered during hospitalization of COVID-19 patients, 41 Brazilian hospitals, March 2020–August 2022 | | | | | |
| --- | --- | --- | --- | --- | --- |
| **Therapies** | **1st wave**  ***n (%)*** | | **2nd wave**  ***n (%)*** | **3rd wave**  ***n (%)*** | ***P-value*** |
| ***n*** | **6799** | | **10,089** | **1474** |  |
| Anticoagulant | 6076^a^ (89.5%) | | 9269^b^ (92.1%) | 1216^c^ (82.6%) | <0.001 |
| Prophylactic anticoagulant dose |  | |  |  |  |
| *Unfractionated heparina* | 1957^a^ (28.8%) | | 2649^b^ (26.3%) | 272^c^ (18.5%) | <0.001 |
| *Low molecular weight heparin* | 3718^a^ (54.7%) | | 6293^b^ (62.4%) | 906^b^ (62.5%) | <0.001 |
| *Fondaparinux* | 64^a^ (0.9%) | | 106^a^ (1.1%) | 0^b^ (0.0%) | <0.001 |
| *Warfarin* | 102^a^ (1.3%) | | 161^a^ (1.4%) | 45^a^ (2.0%) | 0.056 |
| Therapeutic anticoagulant dose |  | |  |  |  |
| *Unfractionated heparina* | 272^a^ (4.0%) | | 371^a^ (3.7%) | 18^b^ (1.2%) | <0.001 |
| *Low molecular weight heparin* | 937^a^ (12.2%) | | 1763^b^ (15.7%) | 221^c^ (9.6%) | <0.001 |
| *Fondaparinux* | 36^a^ (0.5%) | | 17^b^ (0.2%) | 0^b^ (0.0%) | <0.001 |
| Corticoid |  | |  |  |  |
| *Inhalatory corticoid* | | NA | 1391^a^ (13.9%) | 243^a^ (16.5%) | 0.013 |
| *Oral or venous corticoid* | | 4776^a^ (70.4%) | 9311^b^ (92.5%) | 113^c^ (75.6%) | <0.001 |
| *Dexamethasone* | | 3986^a^ (41.3%) | 8569^b^ (85.0%) | 950^c^ (64.5%) | <0.001 |
| *Hydrocortisone* | | 1155^a^ (17.0%) | 1191^b^ (11.8%) | 145^c^ (9.8%) | <0.001 |
| *Metilprednisolone* | | NA | 694^b^ (6.9%) | 70^c^ (4.8%) | <0.001 |
| *Prednisone or prednisolone* | | NA | 1263^b^ (12.5%) | 195^b^ (13.2%) | 0.729 |
| Immunoglobulin | 5^a^ (0.1%) | | 13^a^ (0.1%) | 0^a^ (0.0%) | 0.242 |
| Convalescent plasm | 91^a^ (1.3%) | | 3^b^ (0.0%) | 0^b^ (0.0%) | <0.001 |
| Remdesivir | 6^a^ (0.1%) | | 2^a^ (0.0%) | 0^a^ (0.0%) | 0.028 |
| Sarilumab | 3^a^ (0.0%) | | 3^a^ (0.0%) | 0^a^ (0.0%) | 0.345 |
| Tocilizumab | 7^a^ (0.1%) | | 147^b^ (1.5%) | 11^c^ (0.7%) | <0.001 |
| ECMO | 9^a^ (0.1%) | | 5^a^ (0.0%) | 8^b^ (0.5%) | 0.042 |
| Spontaneous prone position (not intubated) | 1247^a^ (18.3%) | | 1607^b^ (15.9%) | 120^c^ (8.1%) | <0.001 |
| Non-invasive mechanical ventilation | 934^a^ (13.7%) | | 1680^b^ (16.7%) | 112^c^ (7.6%) | <0.001 |
| Numbers are presented as n (%), compared by Pearson's Chi-squared test or Fisher's exact test, if necessary, followed by Z test of proportion comparison with Bonferroni’s correction. ^a,b,c^ If the *P*-value is significant, the superscript letters “a”, “b” and “c” inform in which comparison there is difference. If both groups have the same superscript letter, there is not statistically significance in that comparison. When each group has a different letter, there is a significant difference.  NA: not applicable  1st wave:10 March 2020 to 14 November 2020; 2nd wave: 15 November 2020 to 25 December 2021; 3rd wave: 26 December 2021 to 03 August 2022.  **Table S3.** Vaccination status of COVID-19 patients, 41 Brazilian hospitals, November 2020–August 2022   \| **Characteristics** \| **2nd wave**  ***n (%)*** \| **3rd wave**  ***n (%)*** \| ***P-value*** \| \| --- \| --- \| --- \| --- \| \| ***n*** \| **10,078** \| **1474** \|  \| \| Vaccinated \| 998^b^ (9.9%) \| 677^c^ (46%) \| <0.001 \| \| Missings \| 7243^b^ (72%) \| 676 ^c^ (45.9%) \| <0.001 \| \|  \| **Non-missing cases (*n=998*)** \| **Non-missing cases (*n=677*)** \| <0.001 \| \| Doses \|  \|  \|  \| \| *1* \| 424^b^ (42.5%) \| 29^c^ (4.3%) \|  \| \| *2* \| 493^b^ (49.4%) \| 290^c^ (43.0%) \|  \| \| *3* \| 22^b^ (2.2%) \| 254^c^ (37.6%) \|  \| \| *No information* \| 58^b^ (5.8%) \| 102^c^ (15.1%) \|  \| \| Vaccine brand \|  \|  \|  \| \| *Astrazeneca* \| 216^b^ (21.6%) \| 72^c^ (10.6) \| <0.001 \| \| *CoronaVac* \| 454^b^ (45.5%) \| 103^c^ (15.2%) \| <0.001 \| \| *Janssen* \| 8^b^ (0.8%) \| 3^c^ (0.44%) \| 0.007 \| \| *Pfizer* \| 59^b^ (5.9%) \| 133^c^ (11.5%) \| <0.001 \| \| *Other* \| 2^a^ (0.0%) \| 1^a^ (0.0%) \| 0.167 \|   Numbers are presented as n (%), compared by Pearson's Chi-squared test or Fisher's exact test, if necessary, followed by Z test of proportion comparison with Bonferroni’s correction. ^a,b,c^ If the *P*-value is significant, the superscript letters “a”, “b” and “c” inform in which comparison there is difference. If both groups have the same superscript letter, there is not statistically significance in that comparison. When each group has a different letter, there is a significant difference.  2nd wave: 15 November 2020 to 25 December 2021; 3rd wave: 26 December 2021 to 03 August 2022. | | | | | |

| **Table S4.** Overview of COVID-19 vaccines used in Brazil during the study period | | | |
| --- | --- | --- | --- |
| **Vaccine** | **Manufacturer** | **Type** | **Doses (intervals)** |
| CoronaVac | Sinovac Biotech | Inactivated virus | 2 doses (2–4 weeks apart) |
| ChAdOx1 nCov-19 | AstraZeneca/Oxford University | Viral vector | 2 doses (4–12 weeks apart) |
| BNT162b2 | Pfizer/BioNTech | mRNA | 2 doses (3 weeks apart) |
| Ad26.COV2.S | Janssen / Johnson & Johnson | Viral vector | Single dose (1 dose primary schedule) |

mRNA: messenger RNA

Source: Ministério da Saúde, Governo do Brasil. Plano Nacional de Operacionalização da Vacinação contra a Covid-19 (PNO). Brasília: Ministério da Saúde; 2021.

**Table S5.** Hospitals included in the analysis

| **Health center** | **Funding** | **City** | **State** |
| --- | --- | --- | --- |
| Hospital Bruno Born | Private | Lajeado | Rio Grande do Sul |
| Hospital Cristo Redentor | Public | Porto Alegre | Rio Grande do Sul |
| Hospital da Rede Mater Dei – Betim | Private | Betim | Minas Gerais |
| Hospital da Rede Mater Dei – Contorno | Private | Betim | Minas Gerais |
| Hospital da Rede Mater Dei - Santo Agostinho | Private | Belo Horizonte | Minas Gerais |
| Hospital das Clínicas da Faculdade de Medicina de Botucatu | Public | Botucatu | São Paulo |
| Hospital das Clínicas da Universidade Federal da Pernambuco | Public | Recife | Pernambuco |
| Hospital das Clínicas da Universidade Federal de Minas Gerais | Public | Belo Horizonte | Minas Gerais |
| Hospital de Clínicas de Porto Alegre | Public | Porto Alegre | Rio Grande do Sul |
| Hospital Eduardo de Menezes | Public | Belo Horizonte | Minas Gerais |
| Hospital João XXIII | Public | Belo Horizonte | Minas Gerais |
| Hospital Júlia Kubitschek | Public | Belo Horizonte | Minas Gerais |
| Hospital Luxemburgo | Public | Belo Horizonte | Minas Gerais |
| Hospital Mãe de Deus | Private | Porto Alegre | Rio Grande do Sul |
| Hospital Márcio Cunha | Private | Ipatinga | Minas Gerais |
| Hospital Metropolitano Dr. Célio de Castro | Public | Belo Horizonte | Minas Gerais |
| Hospital Metropolitano Odilon Behrens | Public | Belo Horizonte | Minas Gerais |
| Hospital Moinhos de Vento | Private | Porto Alegre | Rio Grande do Sul |
| Hospital Nossa Senhora da Conceição | Public | Porto Alegre | Rio Grande do Sul |
| Hospital Regional Antônio Dias | Public | Patos de Minas | Minas Gerais |
| Hospital Regional de Barbacena Dr. José Américo | Public | Barbacena | Minas Gerais |
| Hospital Regional do Oeste | Public | Chapecó | Santa Catarina |
| Hospital Risoleta Tolentino Neves | Public | Belo Horizonte | Minas Gerais |
| Hospital Santa Cruz | Private | Santa Cruz do Sul | Rio Grande do Sul |
| Hospital Santa Rosália | Private | Teófilo Otoni | Minas Gerais |
| Hospital Santo Antônio | Private | Curvelo | Minas Gerais |
| Hospital São João de Deus | Private | Divinópolis | Minas Gerais |
| Hospital São Lucas da PUCRS | Public | Porto Alegre | Rio Grande do Sul |
| Hospital Semper | Private | Belo Horizonte | Minas Gerais |
| Hospital SOS Cárdio | Private | Florianópolis | Santa Catarina |
| Hospital Tacchini | Private | Bento Gonçalves | Rio Grande do Sul |
| Hospital Unimed-BH | Private | Belo Horizonte | Minas Gerais |
| Hospital Universitário Canoas | Public | Canoas | Rio Grande do Sul |
| Hospital Universitário Ciências Médicas | Public | Belo Horizonte | Minas Gerais |
| Hospital Universitário Oswaldo Cruz | Public | Recife | Pernambuco |
| Hospital Universitário Professor Edgard Santos | Public | Salvador | Bahia |
| Hospital Universitário Santa Maria | Public | Santa Maria | Rio Grande do Sul |
| Instituto Mario Penna | Public | Belo Horizonte | Minas Gerais |
| Orizonti - Instituto de Saúde e Longevidade Ltda | Private | Belo Horizonte | Minas Gerais |
| Pronto Socorro Cardiológico Universitário de Pernambuco (PROCAPE) | Public | Recife | Pernambuco |
| Santa Casa de Misericórdia de Belo Horizonte | Public | Belo Horizonte | Minas Gerais |
